# Genome Sequences of *Cupriavidus metallidurans* Strains NA1, NA4, and NE12, Isolated from Space Equipment

**DOI:** 10.1128/genomeA.00719-14

**Published:** 2014-07-24

**Authors:** Pieter Monsieurs, Kristel Mijnendonckx, Ann Provoost, Kasthuri Venkateswaran, C. Mark Ott, Natalie Leys, Rob Van Houdt

**Affiliations:** a Unit of Microbiology, Belgian Nuclear Research Centre (SCK•CEN), Mol, Belgium; b Biotechnology and Planetary Protection Group, Jet Propulsion Laboratory, California Institute of Technology, Pasadena, California, USA; c Biomedical Research and Environmental Science Division, NASA, Johnson Space Center, Houston, Texas, USA

## Abstract

*Cupriavidus metallidurans* NA1, NA4, and NE12 were isolated from space and spacecraft-associated environments. Here, we report their draft genome sequences with the aim of gaining insight into their potential to adapt to these environments.

## GENOME ANNOUNCEMENT

*Cupriavidus metallidurans* strains have previously been isolated from industrial sites linked to mining, metallurgic, and chemical industries (1–3) and are characterized by multiple metal resistances (1, 4–8). A substantial portion of the metal-resistance determinants are carried by native megaplasmids such as pMOL28 and pMOL30 of the type strain CH34 (6, 9, 10). In addition, some *C. metallidurans* strains carry an extensive mobile gene pool (5, 11, 12). *C. metallidurans* NA1, NA4, and NE12 were isolated from water samples of the potable water system and the condensate water regeneration system of the International Space Station and from air particulates of the Payload Hazardous Servicing Facility located at the Kennedy Space Center in Florida, respectively (4, 13, 14). These isolates were sequenced for comparative analyses and for exploring the adaptation potential to these oligotrophic environments.

Whole-genome shotgun sequencing and paired-end sequencing of *C. metallidurans* NA4 and NE12 were performed by Macrogen (Seoul, South Korea) using the 454 GS-FLX sequencing platform. The sequencing data for strain NA4 and strain NE12 showed average read lengths of 307 nucleotides (nt) and 333 nt, average insert sizes of 2,972 nt and 2,777 nt, and total sequencing data of 258 Mbp and 409 Mbp, respectively. Both genomes were assembled using Newbler software (version 2.3), resulting in 109 and 87 contigs and *N*_50_ values of 226,033 nt and 197,312 nt for NA4 and NE12, respectively. Full-genome sequencing of *C. metallidurans* NA1 was performed by Baseclear (Leiden, The Netherlands) on the Illumina HiSeq 2000 platform using a combination of a paired-end sequencing library (101 nucleotides) with an average insert size of 240 nt and a mate-pair sequencing library (51 nucleotides) with an average insert size of 3,233 nt. Reads were filtered to remove low-quality reads, resulting in a total of 5,832,127 paired reads (594 Mbp) and 9,473,070 mate-pair reads (1,913 Mbp). *De novo* genome assembly based on the paired-end reads was performed using Velvet (15), resulting in 216 contigs and an *N*_50_ value of 69,748 nt. Applying the scaffolding algorithm SSPACE (16) using the mate-pair library resulted in 27 scaffolds.

The genome of *C. metallidurans* NA1 was estimated to be 6,833,318 bp, with a G+C content of 63.76%. The genome of NA4 was estimated to be 7,370,364 bp, with a G+C content of 63.27%. The genome of NE12 was estimated to be 7,132,975 bp, with a G+C content of 63.64%.

Next to the chromosome and the chromid (17), all three strains carry a megaplasmid comparable in size to pMOL30 (~234 kb). Strains NA1 and NA4 carry a second megaplasmid comparable in size to pMOL28 (~172 kb) (4). Strain NA4 carries an additional plasmid of around 95 kb (4).

The NA1 genome annotation through the MicroScope platform (18) displayed 6,815 coding sequences (CDS), of which 75.6% were classified in at least one cluster of orthologous groups (COG). NA4 displayed 7,467 CDS, of which 70.9% were classified in at least one COG. NE12 displayed 7,026 CDS, of which 74.3% were classified in at least one COG.

### Nucleotide sequence accession numbers.

These whole-genome shotgun projects have been deposited at DDBJ/EMBL/GenBank under the accession numbers JFZD00000000 (version JFZD01000000), JFZE00000000 (version JFZE01000000), and JFZF00000000 (version JFZF01000000) for strains NA1, NA4 and NE12, respectively.
